# Prognostic efficacy of the combination of the pretreatment systemic Immune-Inflammation Index and Epstein-Barr virus DNA status in locally advanced Nasopharyngeal Carcinoma Patients

**DOI:** 10.7150/jca.52539

**Published:** 2021-02-22

**Authors:** Ying Xiong, Liang-Liang Shi, Li-Sheng Zhu, Qian Ding, Li Ba, Gang Peng

**Affiliations:** Cancer Center, Union hospital, Tongji Medical College, Huazhong University of Science and Technology, Wuhan 430022, China.

**Keywords:** systemic immune-inflammation index, Epstein-Barr virus DNA, locally advanced nasopharyngeal carcinoma, prognosis

## Abstract

**Background:** The systemic immune-inflammation index (SII) and Epstein-Barr virus DNA (EBV DNA) levels has been used as a prognostic marker for nasopharyngeal carcinoma (NPC) patients, but there is no in-depth study in locally advanced NPC patients and no research on the predictive value of their combination. Our study aimed to evaluate the prognostic efficacy of the pretreatment SII, EBV DNA levels and their combination in locally advanced NPC patients receiving induction chemotherapy (IC) followed by concurrent chemoradiotherapy (CCRT).

**Materials and methods:** 319 patients diagnosed with locally advanced NPC receiving IC followed by CCRT were retrospectively reviewed (213 in the training cohort and 106 in the validation cohort). The cut-off value for the SII was determined using receiver operating characteristic (ROC) curve. Correlations between characteristics of patients were assessed using the Pearson correlation coefficient. Survival curves for the SII, EBV DNA levels and their combination were analyzed using the Kaplan-Meier method and compared using the log-rank test. Univariate and multivariate analyses were performed by the Cox proportional hazards regression model to evaluate the prognostic impact on overall survival (OS) and progression-free survival (PFS). A prognostic nomogram was generated and its prediction ability was measured by the concordance index (C-index).

**Results:** The optimal cutoff point for the SII was 402.10. A higher SII and EBV DNA positivity were demonstrated to be related to poorer survival outcomes (*P* < 0.05). Multivariate analyses showed that a higher SII, EBV DNA positivity and their combination were powerful independent risk factors for OS and PFS (*P* < 0.05). The SII - EBV DNA had the largest area under the curve (AUC) compared to either score alone. The incorporation of the SII - EBV DNA into established nomogram achieved higher C-index in the prediction of OS and PFS, indicating its superior for predicting survival. All results were found in the training cohort and confirmed in the validation cohort.

**Conclusions:** The pretreatment SII and EBV DNA levels are promising factors for predicting survival in locally advanced NPC patients. The combination of them, which was superior to either score alone, was a complement to the conventional TNM staging system.

## Introduction

Nasopharyngeal carcinoma (NPC), a malignant disease derived from epithelial cells of the nasopharynx, is the 23rd most prevalent cancer, with 129,000 new cases diagnosed worldwide 1. Due to its unique anatomical location and high sensitivity to irradiation, for locally advanced NPC, concurrent chemoradiotherapy (CCRT) was considered the standard treatment 2 in the past. With the improvement of radiation technology, intensity-modulated radiotherapy (IMRT) has become the dominant technique; however, the most common complications are still local recurrence and distant metastasis 3. Increasing clinical evidence supports that induction chemotherapy (IC) can contribute to controlling and abolishing subclinical micro metastasis 4; thus, the use of IC followed by CCRT is recommended to further reduce distant metastasis risk 5. At present, TNM stage is still the gold standard used to predict the prognosis of NPC, but patients with the same stage and the same pathological type often have different prognoses after receiving similar treatment, as the TNM staging system does not take some promising prognostic factors into consideration.

Recently, accumulating evidence has supported that systemic inflammation plays an important role in the development of cancers, including tumor growth, progression and metastasis 6, 7. Inflammation-based indexes such as the platelet to lymphocyte ratio (PLR), neutrophil to lymphocyte ratio (NLR), and monocyte to lymphocyte ratio (MLR) 8, C-reactive protein (CRP) 9, the CRP to Alb ratio (CAR) 10 and two novel factors, a combination of albumin and the lymphocyte count in the form of the prognostic nutritional index (PNI) and a combination of the neutrophil, platelet, and lymphocyte counts in the form of the systemic immune-inflammation index (SII) 8 have been identified as prognostic biomarkers in a variety of cancers, including esophageal cancer, gastric cancer, colorectal cancer, hepatocellular cancer, lung cancer and NPC 11. However, in a previous study on systemic inflammation in NPC, patients at stages I-IV received different treatment regimens; thus, it was difficult to correctly analyze the impact of these indexes. In our study, to eliminate this therapeutic heterogeneity, the prognostic value of the SII in newly diagnosed locally advanced NPC patients at stage III-IVA receiving definitive IC followed by CCRT was studied. Most noteworthy, Epstein-Barr virus DNA (EBV DNA) is considered an effective biomarker that contributes to the pathogenesis and metastasis of NPC, and patients with pretreatment plasma EBV DNA positivity may have a worse prognosis than those with EBV DNA negativity 12. Furthermore, we firstly researched the combined prognostic value of the SII and EBV DNA status in locally advanced NPC, which was regarded as a complement to the conventional TNM staging system, to improve survival prediction and guide appropriate treatment plans for NPC patients.

## Materials and methods

### Patients and study design

Between January 2013 and December 2017, the medical records of 319 patients diagnosed with locally advanced NPC receiving IC followed by CCRT at Union Hospital Cancer Center were retrospectively reviewed. The patients were randomly assigned into two groups: the training cohort of 213 patients and the validation cohort of 106 patients. The inclusion criteria were as follows: 1) age ≥16 years but ≤ 70 years; 2) pathological diagnosis of NPC; 3) Karnofsky performance score (KPS) ≥70; 4) detailed medical records, including nasopharyngeal speculum, contrast-enhanced MRI of the nasopharynx and neck, chest CT, abdominal ultrasonography and whole-body bone scan for staging data, as well as a final diagnosis of re-staged III-IVA NPC based on the 8th edition of the AJCC staging system; 5) no history of anticancer therapy; 6) completion of prescribed IC and CCRT; and 7) complete data of hematological parameters, including simultaneous plasma EBV DNA levels and neutrophil, lymphocyte and platelet counts within 1 week before therapy. The exclusion criteria were as follows: 1) evidence of concomitant tumors at diagnosis; 2) insufficient heart, lung, liver and renal function and 3) severe anemia, acute infection or autoimmune diseases. Written consent was obtained from all enrolled patients and the study was approved by Cancer center of Union hospital of Tongji medical college of Huazhong university of science and technology.

### Methods

Based on our institution guidelines, those patients with stage III or IVA disease were prescribed IC followed by CCRT. The total prescribed IMRT dose was 70Gy/33F to the gross tumor volume of the nasopharynx (GTVnx), 68Gy/33F to the gross tumor volume of the positive neck lymph nodes (GTVnd), and 66Gy/33F to the high-risk sites of microscopic extension defined as clinical target volume 1 (CTV1), and 60Gy/33F to the clinical target volume 2 (CTV2). PTVs were delineated by adding 5 mm and 3 mm to the GTV and CTV, respectively. The fractionated dose was 1.8 to 2.2 Gy at 1 fraction per day on 5 days per week. The regimens of IC were as follows: 1) TPF regimen: docetaxel (75 mg/m^2^/day, day 1), cisplatin (75 mg/m^2^/day, day 1), and 5-fluorouracil (750 mg/m^2^/day, day 1-5); and 2) TP regimen: docetaxel (75 mg/m^2^/day, day 1) and cisplatin (75 mg/m^2^/day, day 1). IC drugs were prescribed every 3 weeks. Moreover, concurrent chemotherapy consisted of cisplatin sensitization with a total dose of 200 mg/m^2^.

### Data collection and clinical endpoints

All peripheral blood was collected in EDTA anticoagulant test tubes and tested for neutrophil, lymphocyte, platelet count and plasma EBV DNA within 1 week before therapy. The definition of the SII is described as follows: SII = total platelet count (10^9^/L) ×total neutrophil count (10^9^/L) / total lymphocyte count (10^9^/L). The BamHI-W region of the EBV genome showed a strong correlation with EBV DNA levels using polymerase chain reaction (PCR). Real-time quantitative PCR was carried out using an EBV PCR quantitative diagnostic kit (Shengxiang Biotechnology, Hunan, China) and a Stratagene Mx3000P analyzer (Agilent Technologies, Germany), and an EBV DNA level of ≥ 400 copies/mL was defined as positive.

The primary outcome of this study was overall survival (OS), which was defined as the time between first treatment and death or last follow-up. While the secondary outcome was progression-free survival (PFS), it was defined as the time that had elapsed between initial treatment and the date of disease progression or death from any cause.

### Follow-up

All patients were assessed every 3 months for the first 2 years after completion of prescribed treatment, every 6 months between the third to fifth year, and then annually thereafter. A complete physical examination, including a nasopharyngeal speculum, contrast-enhanced MRI of the nasopharynx and neck, chest CT, abdominal ultrasonography, and a whole-body bone scan, was performed semiannually. The latest follow-up was conducted at the end of January 2020. All patients were followed up by regular records of each clinic recheck or phone calls.

### Statistical analysis

All the analyses were conducted using SPSS 25.0, GraphPad Prism 8.0 and R software v4.0.3. The cut-off value of the SII was determined using receiver operating characteristic (ROC) curve analyses. The chi-squared test was used to explore the baseline balance between the low and high SII groups. Correlations between variables were assessed using the Pearson correlation coefficient. Survival curves were analyzed using the Kaplan-Meier method and compared using the log-rank test. Univariate and multivariate Cox proportional hazards regressions were conducted to evaluate the prognostic significance of each variable with respect to OS and PFS. The nomogram was explored by the “rms” package of R v4.0.3 software and the concordance index (C-index) was calculated to predict the performance of the established nomogram model. A two-tailed *P* value less than 0.05 was considered statistically significant.

## Results

### Basic patient characteristics

**Figure 1:**
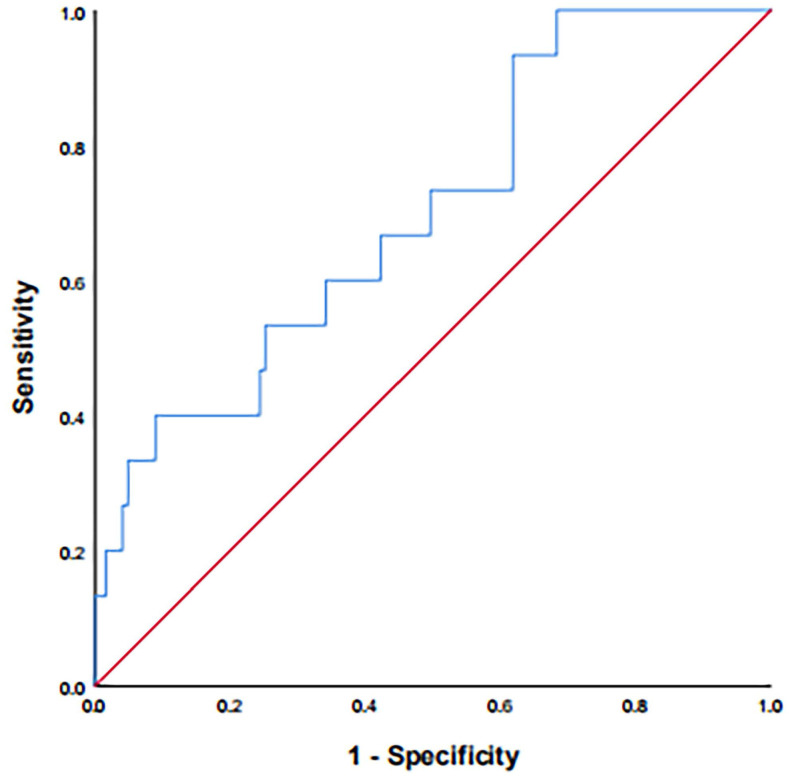
ROC curve for pretreatment SII = 402.10 based on OS. ROC: receiver operating characteristic; SII: systemic immune-inflammation index; OS: overall survival.

A total of 213 and 106 patients were included in the training and validation cohorts, respectively. The cut-off value of the SII was 402.10 according to the ROC curve (Figure 1). The AUC was 0.665 (95% CI: 0.550-0.781, *P* = 0.013, sensitivity: 90.5%, specificity: 39.6%). Based on the cut-off result above, included patients were divided into low and high SII groups. The baseline characteristics of the patients are shown in Table 1. The training cohort consisted of 161 male (75.6%) and 52 females (24.4%), with ages ranging from 24 to 69 years (median 45 years). The latest follow-up was conducted at the end of January 2020. In total, the median follow-up time was 47 months and ranged from 27 months to 83 months. At the end of the study period, 61 (28.6%) patients suffered from tumor progression, and 42 (19.7%) patients died. The validation cohort consisted of 73 male (68.9%) and 33 females (31.1%), with ages ranging from 25 to 67 years (median 47 years). The median follow-up time was 42 months and ranged from 27 months to 82 months. During the follow-up, 20 (18.9%) patients died, 33 (31.1%) suffered from tumor progression.

Table 1 shows the correlations between SII and patient baseline parameters in the two cohorts. There was no correlation between high SII value and gender, age, smoking, drinking, WHO pathological type, EBV DNA, T stage, N stage and IC regimen, which all considered as negative prognostic factors for NPC patients. However, in the validation cohort, SII was significantly correlated with AJCC stage (*P* = 0.034).

### Kaplan-Meier method and log-rank test

**Figure 2:**
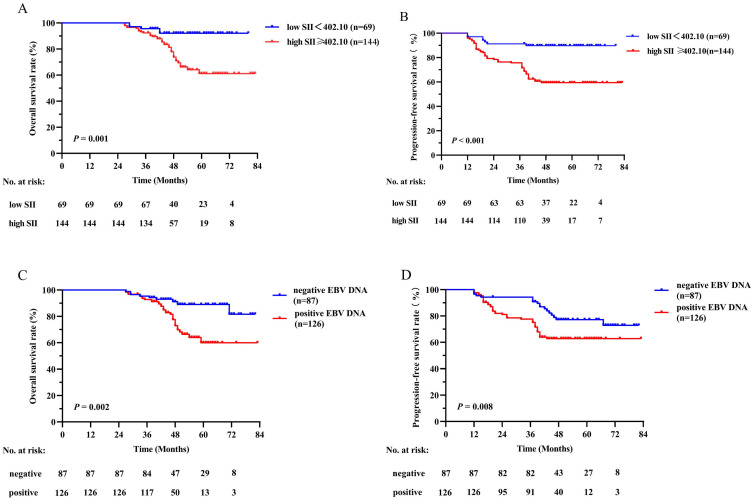
Kaplan-Meier survival curves of OS (A) and PFS (B) between low and high SII groups; OS (C) and PFS (D) between negative and positive EBV DNA groups in the training cohort. Log-rank test, *P* < 0.05 was considered statistically significant. SII: systemic immune-inflammation index; OS: overall survival; PFS: progression-free survival; EBV DNA: Epstein-Barr virus DNA.

**Figure 3:**
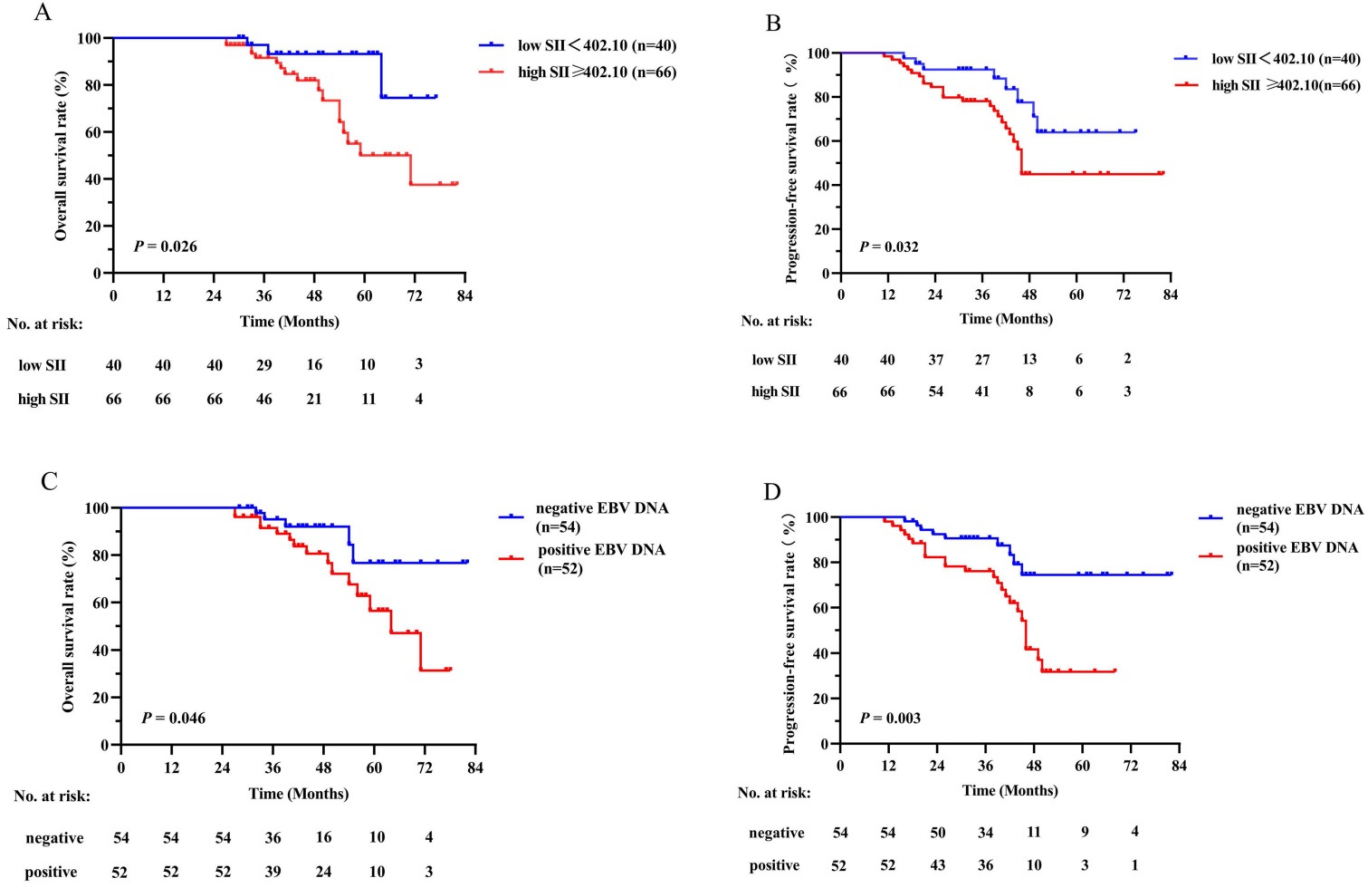
Kaplan-Meier survival curves of OS (A) and PFS (B) between low and high SII groups; OS (C) and PFS (D) between negative and positive EBV DNA groups in the validation cohort. Log-rank test, *P* < 0.05 was considered statistically significant. SII: systemic immune-inflammation index; OS: overall survival; PFS: progression-free survival; EBV DNA: Epstein-Barr virus DNA.

Survival curves based on the SII and plasma EBV DNA status were analyzed using the Kaplan-Meier method and compared using the log-rank test (Figure 2). The survival outcomes in the training cohort revealed that compared with the low SII group, the higher SII group demonstrated poorer OS (*P* = 0.001, Figure 2A) and PFS (*P* < 0.001, Figure 2B). On the basis of different EBV DNA levels, the patients were divided into two groups, with 87 patients in the negative group and 126 patients in the positive group. There was an evident survival difference between the two groups, and patients negative for EBV DNA had superior OS (*P* = 0.002, Figure 2C) and PFS (*P* = 0.008, Figure 2D) compared with patients positive for EBV DNA. These results were confirmed in the validation cohort (Figure 3) (all *P* values < 0.050).

### Univariate and multivariate analysis

In the univariate Cox regression analysis, Tumor classification was corroborated as a potential factor affecting OS in the training cohort (*P =* 0.025) (Table 2). The AJCC stage, SII and EBV DNA were independent predictors of OS and PFS in the training cohort and validation cohort. Variables that reached a significant difference in the univariate analysis were further analyzed considering the influence of confounding factors. Therefore, three separate multivariate models (based on EBV DNA status, AJCC stage, and the SII) were used in the multivariate Cox regression analysis. The complete results are shown in Table 3. As shown in the table, EBV DNA positivity, patients at stage IVA and a high SII were still found to be independent risk factors in NPC patients for OS and PFS in the training cohort and validation cohort (All *P* values < 0.05).

### Combined prognostic value of the SII and EBV DNA status

**Figure 4:**
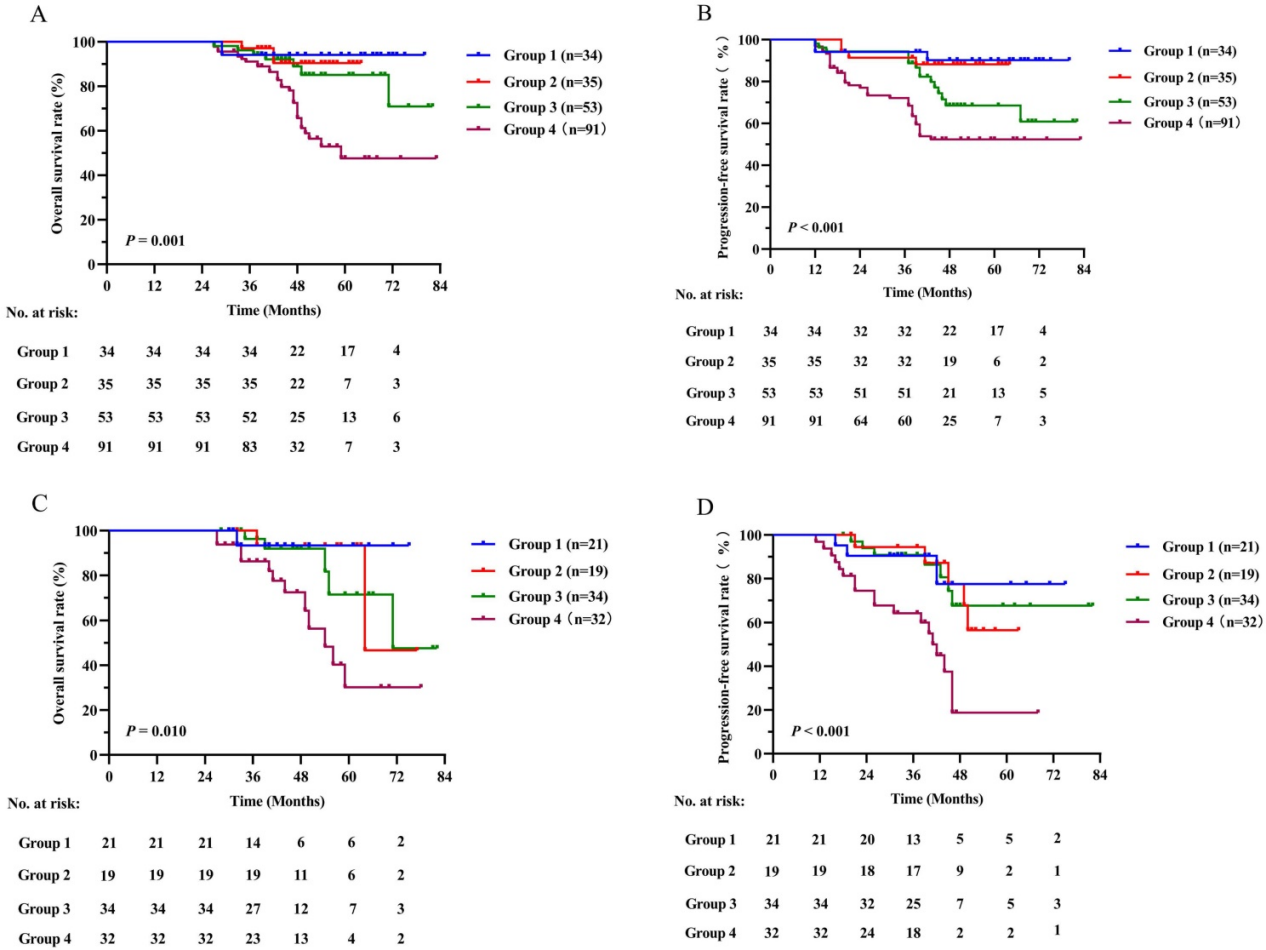
Kaplan-Meier survival curves for OS and PFS between different groups based on SII - EBV DNA status in the training cohort (A, B) and validation cohort (C, D). Log-rank test, *P* < 0.05 was considered statistically significant. SII: systemic immune-inflammation index; OS: overall survival; PFS: progression-free survival; EBV DNA: Epstein-Barr virus DNA.

We further evaluated the predictive value of the combination of SII and EBV DNA status (SII - EBV DNA). The patients were grouped as follows: a low SII and negative EBV DNA group defined as group 1, a low SII and positive EBV DNA group defined as group 2, a high SII and negative EBV DNA defined as group 3, and a high SII and positive EBV DNA group defined as group 4. The number of patients in each group in the training cohort was 34 (16.0%), 35 (16.4%), 53 (24.9%) and 91 (42.7%), respectively. Our results revealed that group 4 had significantly poorer OS and PFS than other groups (*P* = 0.001 and *P* < 0.001 respectively) (Figure 4A, 4B). These results were confirmed in the validation cohort (Figure 4C, 4D).

**Figure 5:**
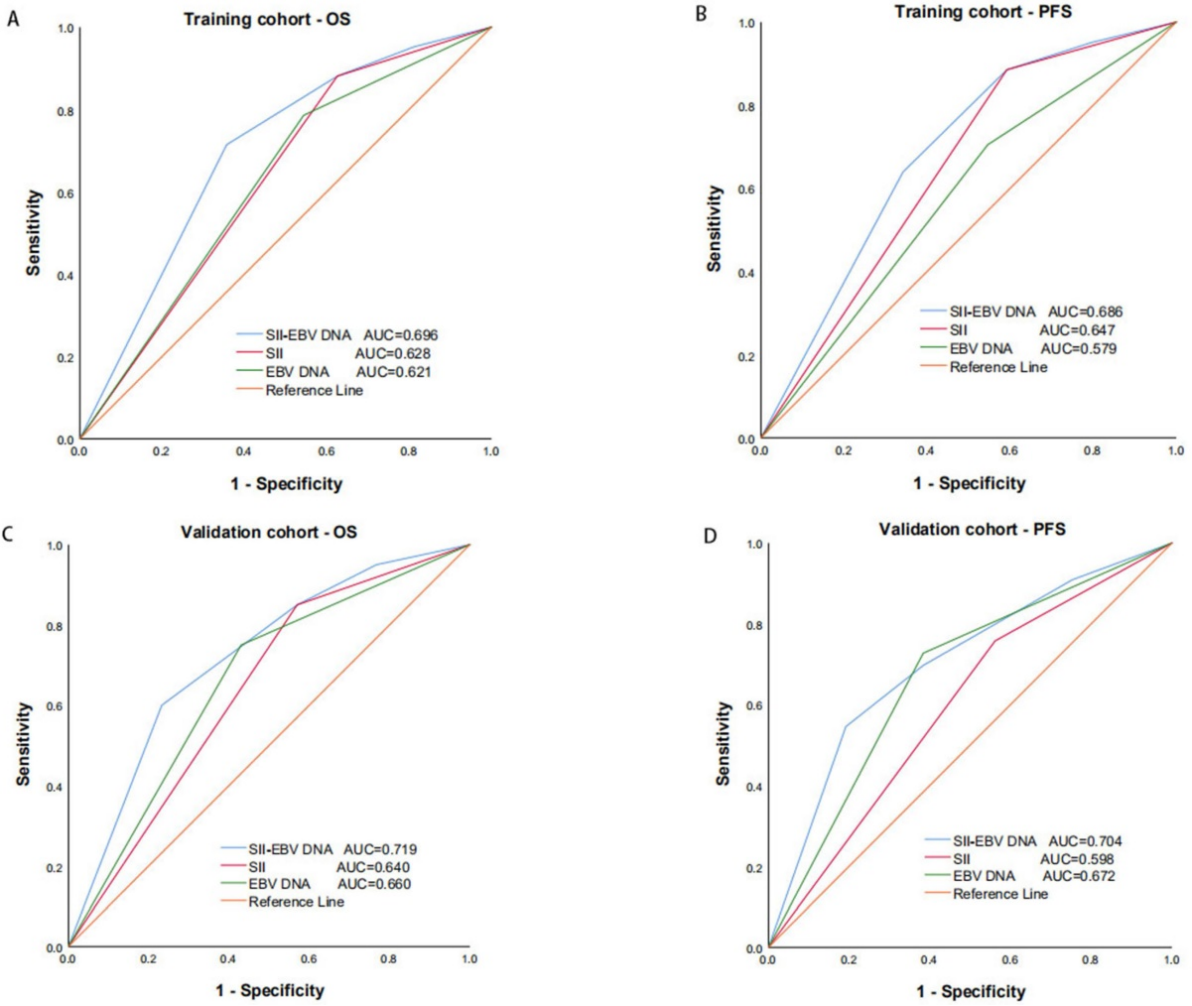
Predictive ability of SII, EBV DNA status and their combination for OS (A) and PFS (B) by ROC curve analysis in training and validation cohorts. SII: systemic immune-inflammation index; EBV DNA: Epstein-Barr virus DNA; OS: overall survival; PFS: progression-free survival; ROC: receiver operating characteristic.

A multivariate analysis was performed to investigate the effects of different SII and EBV DNA status combinations on OS and PFS. Patients with high SII and positive EBV DNA had significantly worse OS and PFS than those with low SII and negative EBV DNA in both the training cohort (OS: HR =7.869; 95% CI: 1.766-35.052; *P* = 0.007; PFS: HR =7.750; 95% CI: 2.208-27.205; *P* = 0.001) and validation cohort (OS: HR =4.000; 95% CI: 1.423-10.188; *P* = 0.022; PFS: HR =7.714; 95% CI: 1.888-31.526; *P* = 0.004) (Table 4). In addition, compared to SII and EBV DNA status alone, the combined SII - EBV DNA achieved the largest AUC in the training cohort and validation cohort by ROC curve (Figure 5), indicating that simultaneously SII and EBV DNA status had better accurate predictive ability for predicting survival and could be identified as a prognostic staging tool for locally advanced NPC patients.

**Figure 6:**
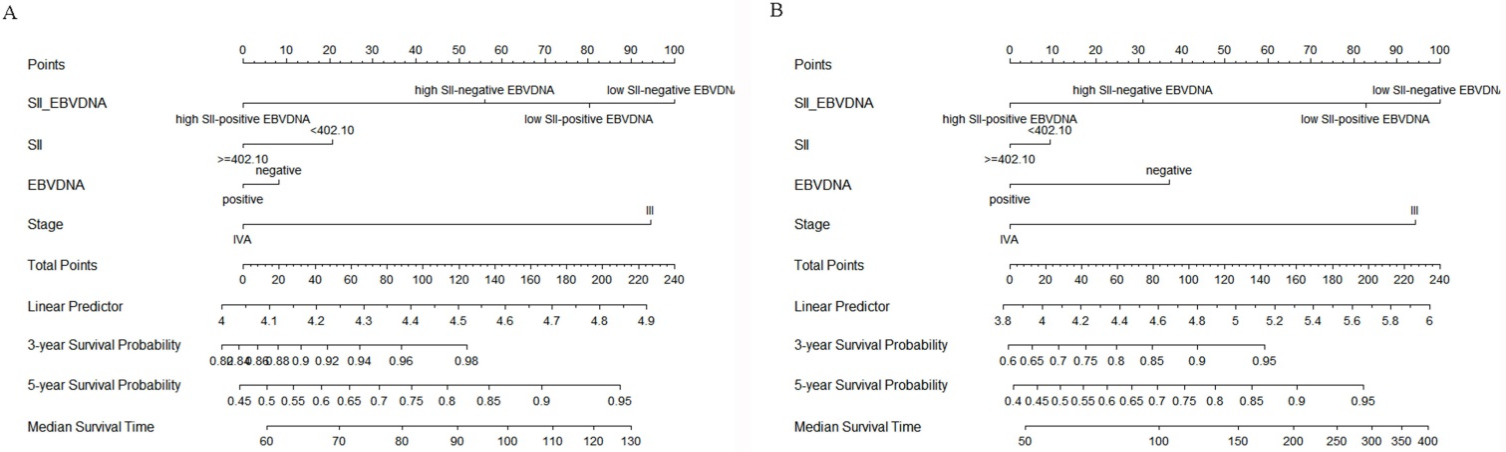
Construction of nomograms to predict OS (A) and PFS (B) in locally advanced NPC patients in the training cohort. SII: systemic immune-inflammation index; EBV DNA: Epstein-Barr virus DNA.

To further predict the survival of NPC patients after IC followed by CCRT, all the significant independent risk prognostic factors of the training cohort were integrated in the nomogram to predict the 3- and 5-year survival using multivariate Cox regression model analysis (Figure 6). The C-indexes in model SII, EBV DNA and SII - EBV DNA for OS prediction were 0.600, 0.596 and 0.657, respectively, which closed corresponded to the actual survival. And also, the C-index in model SII - EBV DNA for PFS prediction was 0.695, higher than others.

## Discussion

In recent years, most NPC patients present at a locally advanced stage when diagnosed and CCRT has become a crucial treatment 2. Studies 13 have shown that IC can control minor subclinical metastases and reduce long-term recurrence, reduce the number of hypoxic cells and increase the sensitivity to radiotherapy to better remove subclinical lesions 14. Therefore, the use of IC followed by CCRT is recommended to further reduce distant metastasis risk. At present, the gold standard used to predict the prognosis of NPC is TNM stage, but patients with the same stage often have different prognosis, as the TNM staging system does not take some potential prognostic factors into consideration. Hence, searching for some prognostic factors is of great clinical value.

Currently, many scholars have realized that systemic inflammation plays an important role in the occurrence, development, infiltration and metastasis of tumors by providing a suitable microenvironment 15. The link between inflammation and cancer can be explained by both intrinsic and extrinsic pathways 16, which interact to initiate the activation of several transcription factors in cancer cells and inflammatory cells, such as nuclear factor-κB and hypoxia-inducible factor 1α (HIF-1α), leading to the formation of a cancer-related inflammatory microenvironment to promote cancer proliferation. In recent years, the role of inflammation related factors including SII, defined as a combination of the neutrophil, platelet and lymphocyte counts, have attracted increased attention as significant predictors for hepatocellular cancer 17, colorectal cancer 18, pancreatic cancer 19 and NPC 11. The prognostic effect of SII in patients with NPC for the first time was studied by Jiang et al, which supporting that the pretreatment SII is an independent predictor for OS in NPC patients 11 and further proved to be superior to the NLR, PLR or MLR as a predictive biomarker. Followed by Oei et al, a total of 585 newly diagnosed NPC patients receiving definitive IMRT-based therapy were reviewed. The results revealed that SII was an independent prognostic factor for OS, PFS, and DMFS 16. In our study, 213 and 106 locally advanced NPC patients receiving IC followed by CCRT in the training and validation cohorts were analyzed retrospectively, which could minimize the treatment regimen bias compared to previous studies. The conclusion of our study demonstrated that a high SII was related to a poorer OS and PFS (all *P* values < 0.05), which is consistent with the previous studies. However, the values of SII were inconsistent in studies, which may be due to the basic level of the included patients with different stages and the difference of sensitivity and reference value of reagent instrument.

The mechanism by which high SII contributes to a poor prognosis in patients with solid cancer is still controversial. Several possible theories can be used to explain the prognostic value of SII by the role of its components. In inflammatory cells, neutrophils can activate endothelial and parenchymal cells, enhance circulating tumor cell adhesion and promote distant metastasis 20. In addition, others explained that neutrophils can inactive T cells and protect cancers cells from immune surveillance 21. Also, it can secrete some inflammatory mediators, such as IL-6 and TNF, to promote NPC cells invasion, proliferation, and metastasis 22. Second, Lymphocytes regulate specific immune responses and control tumor growth by secreting several cytokines, such as IFN-γ and TNF-α 11. And a low level lymphocytes can destroy tumor immune defense by inhibiting cancer cells immune surveillance and blocking cytotoxic cell death, which may produce a favorable tumor microenvironment in the peritoneum for the proliferation, progression and spread of NPC cells 23. Third, platelets can increase the quantity of circulating tumor cells (CTCs) 24 and then induce epithelial-mesenchymal transition (EMT) to promote tumor cell extravasation to metastatic sites. In addition, some evidence suggests that both neutrophils and platelets can contribute to tumor angiogenesis by secreting factors that activate vascular endothelial cells, such as angiopoietin-1 and fibroblast growth factor-2 25. As a result, the combination of high neutrophil count, high platelet count and low lymphocyte count, as defined as a high SII, can promote NPC cell proliferation and metastasis and is related to poor prognosis.

Apart from the SII, EBV DNA status was corroborated as potential factors affecting OS and PFS in our analysis. Some views hold that plasma EBV DNA, as an NPC tumor marker, might originate from active tumor cells 26, such as micro metastatic lesions, which can promote NPC cells reproduction and metastasis, and others hold the opinion that EBV DNA is considered fragments of tumor tissue released directly into the bloodstream 27. In addition, several clinical studies have shown that the level of EBV DNA in NPC patients is related to the number of CTCs, whose internal microenvironment may protect them from immune attack and peripheral blood shear stress, thus promoting metastasis 28. Further, some EBV - encoded oncoprotein like LMP1 and miRNAs could pass through the corresponding cellular signal transduction pathway to mediate EMT of NPC cells, resulting in NPC metastasis 29. Lu et al. retrospectively analyzed 186 new NPC patients and showed that the 5-year OS for patients with high EBV DNA levels was obviously lower than that for patients with low EBV DNA levels 30. Similarly, positive EBV DNA was found to be an independent risk factor in NPC patients for OS and PFS in our cohorts.

To our knowledge, this is the first study of the combined prognostic value of the SII and EBV DNA status in locally advanced NPC. As previously analyzed, a high SII and EBV DNA positivity were independently associated with poorer prognosis in terms of poorer OS and PFS. As we have studied, the combination of higher SII and positive EBV DNA status was a strong independent prognostic factor, which identified patients with higher risk of mortality. Furthermore, through the comparison of binary logistic regression and ROC curve, we found that the combination of them with the largest AUC, was superior to either score alone for predicting OS and PFS. And also, the predictive value of it was also validated in an independent cohort. Most importantly, the predictive accuracy of established nomograms for OS and PFS was improved with increased C-index when incorporating SII - EBV DNA. Based on previous studies, our study made some improvements. The patients included were in a locally advanced stage, and all of them received IMRT and prescribed chemotherapy, which reduced the difference caused by the different treatment methods and enhanced the accuracy and reliability of these parameters. In addition to combining inflammatory indexes with representative markers of EBV DNA status for the first time, we also supplemented the deficiency of previous related studies 16 in terms of the lack of confirmed pathological classification of patients. In our study, no difference in pathological type was shown to affect the prognosis of locally advanced NPC patients (*P* > 0.05). Similarly, Huang et al. found that there was no significant difference in sex, N grade, recurrence, or distant metastasis between WHO type I and WHO type II/III NPC patients 31.

However, our study also has some limitations. First, this was a retrospective study conducted in a single institution, and a relatively large cohort of studies with a longer follow-up period could partly make up for this deficiency. However, another independent cohort was used to validate the results from the training cohort. Second, we only studied the index level before treatment, and analysis of changes in the SII and EBV DNA levels would be more meaningful. Hence, further studies are required in order to validate these preliminary results in locally advanced NPC patients.

## Conclusions

In summary, the pretreatment SII and EBV DNA levels are independent prognostic markers affecting OS and PFS in locally advanced NPC patients diagnosed at stage III-IVA receiving definitive IC followed by CCRT, And the combination of them was superior to either score alone in terms of prognostic ability. Since these hematological indicators are simple, inexpensive, and easily available, combined with conventional TNM staging systems, they can provide a more comprehensive assessment of prognosis and individualized treatment ideas for locally advanced NPC patients.

## Figures and Tables

**Table 1:** Baseline characteristics in training and validation cohorts according to SII

Variables	Training cohort (n = 213)			Validation cohort (n = 106)		
	SII		*P*	SII		*P*
	<402.10 (n=69)	≥402.10 (n=144)		<402.10 (n=40)	≥402.10 (n=66)	
**Gender**			0.282			0.503
female	20	32		14	19	
male	49	112		26	47	
**Age (years)**			0.643			0.258
<60	36	80		21	42	
≥60	33	64		19	24	
**Smoke**			0.067			0.880
no	36	56		20	32	
yes	33	88		20	34	
**Drink**			0.753			0.507
no	38	76		21	39	
yes	31	68		19	27	
**WHO pathological type**			0.187			0.361
I	6	25		4	8	
II	9	22		4	13	
III	54	97		32	45	
**EBV DNA**			0.083			0.803
negative	34	53		21	33	
positive	35	91		19	33	
**Tumor classification**			0.616			0.414
T1-T2	11	27		7	16	
T3-T4	58	117		33	50	
**Nodal classification**			0.735			0.352
N0-N1	13	30		11	13	
N2-N3	56	114		29	53	
**AJCC stage (8^th^)**			0.209			0.034^*^
III	37	64		23	24	
IVA	32	80		17	42	
**IC regimen**						
TP	33	59	0.345	11	29	0.091
TPF	36	85		29	37	

Note: *statistically significant. SII: systemic immune-inflammation index; WHO: World Health Organization; EBV DNA: Epstein-Barr virus DNA; AJCC: American Joint Committee on Cancer; IC: induction chemotherapy.

**Table 2:** Univariate analysis of OS and PFS in training and validation cohorts

Variables	OS		PFS	
	HR (95% CI)	*P*	HR (95% CI)	*P*
**Training cohort**				
Sex (male vs female)	1.125 (0.520-2.435)	0.765	1.679 (0.797-3.535)	0.173
Age (≥60 vs <60 years)	1.293 (0.651-2.566)	0.463	1.567 (0.854-2.878)	0.147
Smoke (yes vs no)	0.870 (0.438-1.729)	0.692	1.670 (0.901-3.094)	0.103
Drink (yes vs no)	0.945 (0.480-1.857)	0.869	1.164 (0.642-2.110)	0.617
**WHO pathological type I**	Ref	Ref	Ref	Ref
(II vs I)	1.967 (0.813-4.757)	0.133	2.209 (1.000-4.883)	0.052
(III vs I)	1.402 (0.547-3.597)	0.482	0.644 (0.246-1.682)	0.369
EBV DNA (positive vs negative)	3.075 (1.387-6.818)	0.006^*^	1.986 (1.051-3.752)	0.035^*^
Tumor classification (T3-4 vs T1-2)	5.333 (1.230-23.125)	0.025^*^	1.976 (0.819-4.768)	0.129
Nodal classification (N2-3 vs N0-1)	0.475 (0.221-1.019)	0.056	0.608 (0.300-1.231)	0.166
AJCC stage (8^th^) (IVA vs III)	5.067 (2.217-11.585)	0.001^*^	5.493 (2.282-8.850)	0.001^*^
SII (≥402.10 vs <402.10)	4.426 (1.655-11.839)	0.003^*^	4.314 (2.269-12.449)	0.001^*^
IC regimen (TPF vs TP)	0.800 (0.406-1.575)	0.519	0.781 (0.430-1.419)	0.417
**Validation cohort**				
Sex (male vs female)	2.035 (0.623-6.645)	0.239	2.055 (0.785-5.379)	0.142
Age (≥60 vs <60 years)	1.251 (0.469-3.338)	0.654	1.339 (0.583-3.077)	0.491
Smoke (yes vs no)	1.222 (0.460-3.247)	0.687	1.034 (0.454-2.353)	0.937
Drink (yes vs no)	1.781 (0.668-4.747)	0.249	1.349 (0.590-3.084)	0.478
**WHO pathological type I**	Ref	Ref	Ref	Ref
(II vs I)	1.538 (0.233-10.153)	0.655	0.429 (0.076-2.419)	0.337
(III vs I)	1.111 (0.219-5.642)	0.899	1.020 (0.281-3.703)	0.976
EBV DNA (positive vs negative)	3.973 (1.325-11.917)	0.014^*^	3.286 (1.743-10.538)	0.002^*^
Tumor classification (T3-4 vs T1-2)	2.908 (0.622-13.583)	0.175	1.833 (0.616-5.453)	0.276
Nodal classification (N2-3 vs N0-1)	0.851 (0.274-2.642)	0.780	0.546 (0.212-1.402)	0.209
AJCC stage (8^th^) (IVA vs III)	4.486 (1.224-16.446)	0.024^*^	4.145 (1.530-11.229)	0.005^*^
SII (≥402.10 vs <402.10)	3.279 (1.167-15.693)	0.028^*^	2.439 (0.971-6.124)	0.048^*^
IC regimen (TPF vs TP)	0.689 (0.257-1.844)	0.458	0.903 (0.388-2.101)	0.813

Note: *statistically significant. SII: systemic immune-inflammation index; WHO: World Health Organization; EBV DNA: Epstein-Barr virus DNA; AJCC: American Joint Committee on Cancer; OS: overall survival; PFS: progression-free survival; HR: hazard ratio; CI: confidence interval; IC: induction chemotherapy.

**Table 3:** Multivariate Cox regression analysis of OS and PFS in training and validation cohorts

Variables	OS		PFS	
	HR (95% CI)	*P*	HR (95% CI)	*P*
**Training cohort**				
EBV DNA (positive vs negative)	1.891(1.874-4.090)	0.015^*^	1.513(1.859-2.664)	0.050^*^
AJCC stage (8^th^) (IVA vs III)	4.963(1.896-9.257)	0.001^*^	4.439(2.268-7.728)	0.001^*^
SII (≥402.10 vs <402.10)	3.732(1.447-9.627)	0.006^*^	3.366(1.976-9.649)	0.001^*^
**Validation cohort**				
EBV DNA (positive vs negative)	3.395(1.851-6.740)	0.018^*^	3.112(1.048-1.905)	0.047^*^
AJCC stage (8^th^) (IVA vs III)	4.972(1.086-13.843)	0.037^*^	4.661(1.172-3.578)	0.012^*^
SII (≥402.10 vs <402.10)	2.643(1.046-12.689)	0.042^*^	1.285(1.737-2.243)	0.036^*^

Note: *statistically significant. SII: systemic immune-inflammation index; EBV DNA: Epstein-Barr virus DNA; AJCC: American Joint Committee on Cancer; OS: overall survival; PFS: progression-free survival; HR: hazard ratio; CI: confidence interval.

**Table 4:** Multivariate analysis of variables for OS and PFS in training and validation cohorts when SII - EBV DNA was incorporated

Variables	OS		PFS	
	HR (95% CI)	*P*	HR (95% CI)	*P*
***Training cohort***				
**SII - EBV DNA**				
Low SII+negative EBV DNA	Ref	Ref	Ref	Ref
Low SII+positive EBV DNA vs Low SII+negative EBV DNA	1.500 (0.235-9.588)	0.668	1.333 (0.275-6.457)	0.721
High SII+negative EBV DNA vs Low SII+negative EBV DNA	2.435 (0.475-12.489)	0.286	4.079 (1.082-15.380)	0.038^*^
High SII+positive EBV DNA vs Low SII+negative EBV DNA	7.869 (1.766-35.052)	0.007^*^	7.750 (2.208-27.205)	0.001^*^
***Validation cohort***				
**SII - EBV DNA**				
Low SII+negative EBV DNA	Ref	Ref	Ref	Ref
Low SII+positive EBV DNA vs Low SII+negative EBV DNA	2.353 (0.196-28.266)	0.500	1.143 (0.436-10.536)	0.348
High SII+negative EBV DNA vs Low SII+negative EBV DNA	3.448 (0.374-31.792)	0.275	3.556 (0.355-6.821)	0.558
High SII+positive EBV DNA vs Low SII+negative EBV DNA	4.000 (1.423-10.188)	0.022^*^	7.714 (1.888-31.526)	0.004^*^

Note: *statistically significant. SII: systemic immune-inflammation index; EBV DNA: Epstein-Barr virus DNA; OS: overall survival; PFS: progression-free survival; HR: hazard ratio; CI: confidence interval.

## Abbreviations

SII: systemic immune-inflammation index
EBV DNA: Epstein-Barr virus DNA
NPC: nasopharyngeal carcinoma
IC: induction chemotherapy
CCRT: concurrent chemoradiotherapy
ROC: receiver operating characteristic
OS: overall survival
PFS: progression-free survival
IMRT: intensity-modulated radiotherapy
PLR: platelet to lymphocyte ratio
NLR: neutrophil to lymphocyte ratio
MLR: monocyte to lymphocyte ratio
CRP: C-reactive protein
CAR: CRP to Alb ratio
PNI: prognostic nutritional index
KPS: Karnofsky performance score
GTVnx: gross tumor volume of the nasopharynx
GTVnd: the gross tumor volume of the positive neck lymph nodes
CTV1: clinical target volume 1
CTV2: clinical target volume 2
PCR: polymerase chain reaction
AUC: area under the ROC curve
WHO: World Health Organization
AC: adjuvant chemotherapy
HIF-1α: hypoxia-inducible factor 1α
CTCs: circulating tumor cells
EMT: epithelial-mesenchymal transition
AJCC: American Joint Committee on Cancer
HR: hazard ratio
CI: confidence interval
Ref: reference
C-index: concordance index

## References

[1] Bray F, Ferlay J, Soerjomataram I, Siegel RL, Torre LA, Jemal A (2018). Global cancer statistics 2018: GLOBOCAN estimates of incidence and mortality worldwide for 36 cancers in 185 countries. CA Cancer J Clin.

[2] Blanchard P, Lee A, Marguet S, Leclercq J, Ng WT, Ma J (2015). Chemotherapy and radiotherapy in nasopharyngeal carcinoma: an update of the MAC-NPC meta-analysis. Lancet Oncol.

[3] Qiu WZ, Peng XS, Xia HQ, Huang PY, Guo X, Cao KJ (2017). A retrospective study comparing the outcomes and toxicities of intensity-modulated radiotherapy versus two-dimensional conventional radiotherapy for the treatment of children and adolescent nasopharyngeal carcinoma. J Cancer Res Clin Oncol.

[4] Liu T, Sun Q, Chen J, Li B, Qin W, Wang F (2018). Neoadjuvant Chemotherapy with Fluorouracil plus Nedaplatin or Cisplatin for Locally Advanced Nasopharyngeal Carcinoma: a Retrospective Study. J Cancer.

[5] Chen YP, Tang LL, Yang Q, Poh SS, Hui EP, Chan ATC (2018). Induction Chemotherapy plus Concurrent Chemoradiotherapy in Endemic Nasopharyngeal Carcinoma: Individual Patient Data Pooled Analysis of Four Randomized Trials. Clin Cancer Res.

[6] Diakos CI, Charles KA, McMillan DC, Clarke SJ (2014). Cancer-related inflammation and treatment effectiveness. Lancet Oncol.

[7] Fernandes JV, Cobucci RNO, Jatobá CAN, Fernandes TAAdM, de Azevedo JWV, de Araújo JMG (2015). The role of the mediators of inflammation in cancer development. Pathol Oncol Res.

[8] Wang L, Wang C, Wang J, Huang X, Cheng Y (2017). A novel systemic immune-inflammation index predicts survival and quality of life of patients after curative resection for esophageal squamous cell carcinoma. J Cancer Res Clin Oncol.

[9] Fang Y, Xu C, Wu P, Zhang LH, Li DW, Sun JH (2017). Prognostic role of C-reactive protein in patients with nasopharyngeal carcinoma: A meta-analysis and literature review. Medicine (Baltimore).

[10] Ishizuka M, Nagata H, Takagi K, Iwasaki Y, Shibuya N, Kubota K (2016). Clinical Significance of the C-Reactive Protein to Albumin Ratio for Survival After Surgery for Colorectal Cancer. Ann Surg Oncol.

[11] Jiang W, Chen Y, Huang J, Xi D, Chen J, Shao Y (2017). Systemic immune-inflammation index predicts the clinical outcome in patients with nasopharyngeal carcinoma: a propensity score-matched analysis. Oncotarget.

[12] Nilsson JS, Forslund O, Andersson FC, Lindstedt M, Greiff L (2019). Intralesional EBV-DNA load as marker of prognosis for nasopharyngeal cancer. Sci Rep.

[13] Ribassin-Majed L, Marguet S, Lee AWM, Ng WT, Ma J, Chan ATC (2017). What Is the Best Treatment of Locally Advanced Nasopharyngeal Carcinoma? An Individual Patient Data Network Meta-Analysis. J Clin Oncol.

[14] He X, Xu K, Guo J, Zhu Y, Liang X, Liu L (2015). A meta-analysis of neoadjuvant chemotherapy plus radiation in the treatment of locally advanced nasopharyngeal carcinoma. J Cancer Res Ther.

[15] Oliva N, Carcole M, Beckerman M, Seliktar S, Hayward A, Stanley J (2015). Regulation of dendrimer/dextran material performance by altered tissue microenvironment in inflammation and neoplasia. Sci Transl Med.

[16] Oei RW, Ye L, Kong F, Du C, Zhai R, Xu T (2018). Prognostic value of inflammation-based prognostic index in patients with nasopharyngeal carcinoma: a propensity score matching study. Cancer Manag Res.

[17] Hu B, Yang XR, Xu Y, Sun YF, Sun C, Guo W (2014). Systemic immune-inflammation index predicts prognosis of patients after curative resection for hepatocellular carcinoma. Clin Cancer Res.

[18] Chen JH, Zhai ET, Yuan YJ, Wu KM, Xu JB, Peng JJ (2017). Systemic immune-inflammation index for predicting prognosis of colorectal cancer. World J Gastroenterol.

[19] Aziz MH, Sideras K, Aziz NA, Mauff K, Haen R, Roos D (2019). The Systemic-immune-inflammation Index Independently Predicts Survival and Recurrence in Resectable Pancreatic Cancer and its Prognostic Value Depends on Bilirubin Levels: A Retrospective Multicenter Cohort Study. Ann Surg.

[20] Gao Y, Guo W, Cai S, Zhang F, Shao F, Zhang G (2019). Systemic immune-inflammation index (SII) is useful to predict survival outcomes in patients with surgically resected esophageal squamous cell carcinoma. J Cancer.

[21] Zheng Y, Yu D, Yu Z, Zhao D, Chen Y, Chen W (2020). Association of preoperative systemic Immune-inflammation Index and Prognostic Nutritional Index with survival in patients with Upper Tract Urothelial Carcinoma. J Cancer.

[22] Tecchio C, Scapini P, Pizzolo G, Cassatella MA (2013). On the cytokines produced by human neutrophils in tumors. Semin Cancer Biol.

[23] Dolan RD, McSorley ST, Horgan PG, Laird B, McMillan DC (2017). The role of the systemic inflammatory response in predicting outcomes in patients with advanced inoperable cancer: Systematic review and meta-analysis. Crit Rev Oncol Hematol.

[24] Mego M, Gao H, Cohen EN, Anfossi S, Giordano A, Tin S (2017). Circulating tumor cells (CTCs) are associated with abnormalities in peripheral blood dendritic cells in patients with inflammatory breast cancer. Oncotarget.

[25] Cools-Lartigue J, Spicer J, McDonald B, Gowing S, Chow S, Giannias B (2013). Neutrophil extracellular traps sequester circulating tumor cells and promote metastasis. J Clin Invest.

[26] Chan KCA, Zhang J, Chan ATC, Lei KIK, Leung SF, Chan LYS (2003). Molecular characterization of circulating EBV DNA in the plasma of nasopharyngeal carcinoma and lymphoma patients. Cancer Res.

[27] Ma BBY, Mo FKF, Chan ATC, Hui EP, Leung SF, Lo YMD (2008). The prognostic significance of tumor vascular invasion and its association with plasma Epstein-Barr virus DNA, tumor volume and metabolic activity in locoregionally advanced nasopharyngeal carcinoma. Oral Oncol.

[28] He C, Huang X, Su X, Tang T, Zhang X, Ma J (2017). The association between circulating tumor cells and Epstein-Barr virus activation in patients with nasopharyngeal carcinoma. Cancer Biol Ther.

[29] Yang Z, Wang J, Zhang Z, Tang F (2019). Epstein-Barr Virus-Encoded Products Promote Circulating Tumor Cell Generation: A Novel Mechanism of Nasopharyngeal Carcinoma Metastasis. Onco Targets Ther.

[30] Lu L, Li J, Zhao C, Xue W, Han F, Tao T (2016). Prognostic efficacy of combining tumor volume with Epstein-Barr virus DNA in patients treated with intensity-modulated radiotherapy for nasopharyngeal carcinoma. Oral Oncol.

[31] Guo ZM, Liu WW, He JH (2009). A retrospective cohort study of nasopharyngeal adenocarcinoma: a rare histological type of nasopharyngeal cancer. Clin Otolaryngol.

